# A rapid two-dimensional data collection system for the study of ferroelectric materials under external applied electric fields

**DOI:** 10.1107/S1600576716011341

**Published:** 2016-08-16

**Authors:** Tikhon Vergentev, Iurii Bronwald, Dmitry Chernyshov, Semen Gorfman, Stephanie H. M. Ryding, Paul Thompson, Robert J. Cernik

**Affiliations:** a Peter the Great Polytechnic University, St Petersburg, Russian Federation; b ESRF, 71 Rue des Martyrs, Grenoble, France; c Department of Physics, University of Siegen, Siegen, Germany; d School of Materials, The University of Manchester, Oxford Road, Manchester M13 9PL, UK

**Keywords:** ferroelectricity, piezoelectricity, diffuse scattering, *in situ* two-dimensional X-ray single-crystal diffraction, area detectors, synchrotron X-rays, PMN-PT, phase transitions

## Abstract

Synchrotron X-rays on the Swiss Norwegian Beamline and BM28 (XMaS) at the ESRF have been used to record the diffraction response of the PMN–PT relaxor piezoelectric 67% Pb(Mg_1/3_Nb_2/3_)O_3_–33% PbTiO_3_ as a function of externally applied electric field. A DC field in the range 0–18 kV cm^−1^ was applied along the [001] pseudo-cubic direction using a specially designed sample cell for *in situ* single-crystal diffraction experiments. The cell allowed data to be collected on a Pilatus 2M area detector in a large volume of reciprocal space using transmission geometry. The data showed good agreement with a twinned single-phase monoclinic structure model. The results from the area detector were compared with previous Bragg peak mapping using variable electric fields and a single detector where the structural model was ambiguous. The coverage of a significantly larger section of reciprocal space facilitated by the area detector allowed precise phase analysis.

## Abstract 2

The diffraction response of the PMN–PT relaxor piezoelectric 67% Pb(Mg_1/3_Nb_2/3_)O_3_–33% PbTiO_3_ has been recorded as a function of externally applied electric field. The DC field was applied using a specially designed sample cell for *in situ* single-crystal diffraction experiments. The coverage of a significantly large section of reciprocal space allowed precise phase analysis.

## 1. Introduction   

Designer ceramic materials are often the first industrial choice for switches, actuators, and piezoelectric, thermoelectric and microwave applications. These materials are often deliberately manufactured with compositions close to a solid state phase transition point or a line in the phase diagram separating ferroelectric phases known as a morphotropic phase boundary (MPB). MPB ferroelectrics show interesting properties that are attractive for both fundamental research and technical applications (Kuwata *et al.*, 1981 ▸, 1982 ▸). Single crystals of these materials show much higher values of dielectric constant and electromechanical coupling factor than those observed for polycrystalline ceramics, as described by Park & Shrout (1997*a* ▸,*b* ▸), Harada *et al.* (1998 ▸) and Luo *et al.* (2000 ▸). The structural reasons for the strong enhancement of dielectric and electromechanical properties are linked to the composite nature of the crystals. Most ferroelectric perovskites near the MPB are mixed crystals with several different polar phases that may coexist in a broad temperature range. For example, 67% Pb(Mg_1/3_Nb_2/3_)O_3_–33% PbTiO_3_ (PMN–33%PT) at tem­peratures above 420 K is a primitive cubic perovskite. In the temperature range 420–380 K it becomes tetragonal, and below 380 K it shows a coexistence of tetragonal and monoclinic structural domains (Araújo, 2011 ▸; Singh *et al.*, 2006 ▸) which react differently when an electric field is applied (Noheda *et al.*, 2001 ▸). Detailed and deliberate manipulation of the domain structure for such composite material is a challenge that is known as ‘domain engineering’ (Aleshin & Raevski, 2014 ▸). It requires a detailed description of various processes occurring in a crystal and evaluation of their contributions to ultrahigh piezoelectric constants and field-induced strains.

*In situ* single-crystal diffraction experiments are regarded as powerful instruments to document evolution of lattice parameters and domain structure (Aleshin & Raevski, 2014 ▸; Fu & Cohen, 2000 ▸; Jo *et al.*, 2011 ▸; Bosak *et al.*, 2015 ▸) and hence can be used to obtain this information to assist domain engineering. Several diffraction experiments under electric fields have been carried out with PMN-based ferroelectric crystals with compositions close to the MPB (Kitanaka *et al.*, 2014 ▸; Levin *et al.*, 2006 ▸; Cao *et al.*, 2006 ▸). For example, an experiment with an alternating electric field applied to a PMN–32%PT crystal was carried out on the XMaS beamline at the ESRF. This experimental approach allowed both the determination of the crystal structure under the electric field and an evaluation of the dynamic ferroelectric response (Wooldridge *et al.*, 2012 ▸).

Electric fields are often applied to plate-shaped crystals *via* metallic contacts that are deposited on the sides of the plate. In this case X-ray diffraction data can be collected in reflection geometry with the X-ray beam transmitted through the top contact. Although it is easier to apply the electric field this way, this experimental geometry is far from ideal from a diffraction point of view. Firstly, only a limited number of Bragg nodes can be accessed and accurately measured; secondly, the rather small penetration depth of X-ray radiation increases the contribution from near surface zones of the crystal. Consequently, the result may not be entirely relevant for the bulk. Finally the measurements with a point detector require considerable counting time to collect reciprocal space maps around selected Bragg peaks. However, high-resolution reciprocal space maps are desirable for resolving diffraction contributions from multiple twin domains, which are inherent to all perovskite-based functional materials.

Significant improvements to the single detector approach have been reported by Daniels *et al.* (2012 ▸, 2011 ▸), where reciprocal space volumes or maps have been collected with an applied electric field using a CCD camera, a purpose built sample cell and high-energy synchrotron radiation on station ID15 at the ESRF, Grenoble. Their impressive results showed short-range structural correlations at the atomic scale and nanometre-sized rhombohedral octahedral tilt domains separated by stacking faults. The electric field application removed these faults from the crystal and resulted in a rhombohedral domain growth. They were also able to measure frequency dependent effects. The crystal samples were quite large (average dimensions 3 × 1 × 1 mm) and mounted in an oil filled cell with limited exit apertures. The approach we describe in this paper uses much smaller crystals in transmission geometry with no limitations on exit aperture and, more importantly, no oil in the cell, which increases the level of background scattering. Our use of the Pilatus 2M *versus* a CCD further improves the signal-to-noise ratio. The PILATUS 2M has a much higher dynamic range (compared with a CCD), does not suffer from overexposure from strong Bragg peaks, and does not distort the weaker scattering signals for diffuse or satellite reflections. This, when coupled with our use of lower energies (smaller crystal samples), allows us to measure the diffuse scattering with far greater precision and could facilitate laboratory-based experiments using our system.

The aim of this work is to demonstrate our new experimental approach which overcomes the difficulties associated with using large plate-shaped single crystals (for the application of electric fields) and the limitations of using point detectors. We have developed a new strategy for a synchrotron X-ray experiment in transmission geometry using an area detector with the sample being subjected to an external electric field. We have used this approach to study the structural response of a ferroelectric near its MPB. A Pilatus@SNBL single-crystal diffractometer equipped with a Pilatus 2M pixel area detector (Dyadkin *et al.*, 2016 ▸) has been used to map the intensity of X-ray scattering. The electric field has been applied using a purpose built cell that allows electrical contacting of tiny (less than 100 µm in diameter) single-crystal rods in a suitable configuration for measurements in transmission geometry (Vergentev *et al.*, 2015 ▸). Diffraction data were recorded for a single crystal of PMN–33%PT, previously studied with a point detector in reflection geometry (Wooldridge *et al.*, 2012 ▸). All the data are consistent with the formation of a dominant monoclinic phase in which the lattice parameters are sensitive to the electric field. The deformation and evolution of the twin pattern were observed to follow the structural processes underlying the intrinsic electromechanical response for this and similar ferroelectrics.

## 2. Experimental   

**Figure 1:**
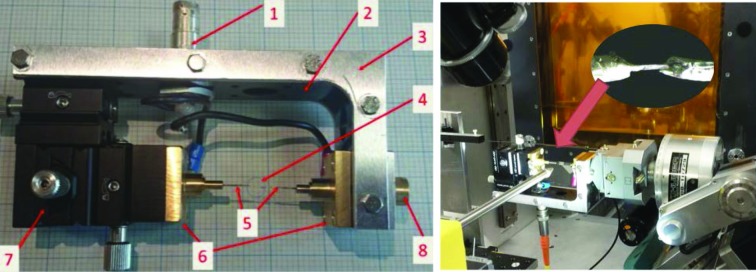
(Left) The sample cell for the application of electric fields in diffraction transmission mode. (1) HV connector; (2) Teflon skeleton; (3) aluminium frame; (4) sample; (5) molybdenum needles; (6) holder; (7) coordinate table; (8) goniometer head mount. (Right) How the cell is mounted on the goniometer head and rotated with a mini-kappa Huber goniometer. The inset shows a single-crystal sample fixed between two needle contacts with conducting glue.

A single crystal of PMN–33%PT was cut and polished down to a rod shape approximately 3 mm in length and 100 µm thick, with the [001] direction along the rod axis. The sample was then boiled for a few minutes in a 20% HCl solution to clean the surface and remove the surface layer damaged by the mechanical treatment. The sample was fixed with silver paint between two electrodes approximately 700 µm apart on the electric cell. A static electric field was applied (along the rod axis [001] direction) and data were collected by rotating the entire cell (using the phi motor) with the rotation axis close to [001]. Sample alignment, rotation and data acquisition were all carried out with the Pilatus@SNBL diffractometer (BM01A station, SNBL at ESRF, Grenoble) (Dyadkin *et al.*, 2016 ▸). The data were recorded by collecting two-dimensional frames (at a rate of two million pixels per frame with a 0.172 mm pixel size) with a 0.1° angular step. The experimental diffraction setup with the electric cell is shown in Fig. 1 ▸.

In order to transform the considerable volumes of data (Tb per experiment) into a usable and interpretable form (*e.g.* reciprocal space maps and crystallographic projections), the following procedure was adopted. The raw Pilatus 2M frames were preprocessed with the *SNBL Toolbox* (Dyadkin *et al.*, 2016 ▸); the orientation matrix and reciprocal space maps were reconstructed with the *CrysAlis* software (Rigaku, 2015 ▸) with the diffracted intensities distributed in a volume near Bragg nodes of the cubic lattice. The position of every volume element (voxel) is given by the scattering vector pointing to the center of the voxel. The length of the scattering vector is defined by the corresponding Bragg angle 2θ with its orientation fixed by two angles, φ and ω. φ is given by the experimental positions of the scanning axis, while 2θ and ω are calculated assuming that the detector plane is normal to the primary X-ray beam: 
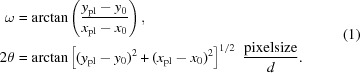
Here ω is the angle of rotation (inclination) of each detector pixel around the primary X-ray beam, *y*_0_ and *x*_0_ are the coordinates of the normal to the detector plane starting from the sample position in the local area detector coordinate system, and *y*_pl_ and *x*_pl_ are each pixel’s coordinates in the local area detector coordinate system. The parameter pixelsize is the size of the pixel, and *d* is the distance from the sample to the detector plane. Integration over φ near Bragg nodes gives high-resolution patterns providing information on the splitting of reflections from different twin domains.

## 3. Results   

**Figure 2:**
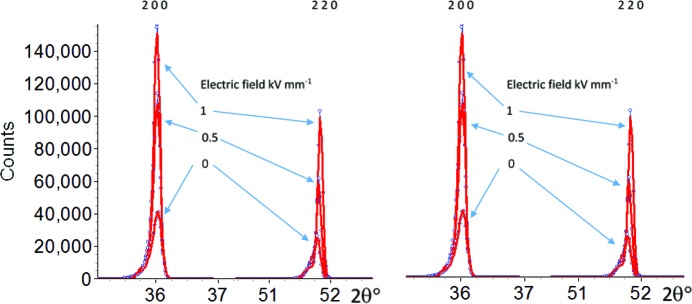
The 200 reflection and 220 reflection from a single-crystal sample of PMN–32%PT. The diagram shows the measured diffraction pattern (blue) together with the calculated fit (red) for both models. The two-phase model is shown on the left, the single phase on the right. Both models are seen to fit well. For clarity only three electric field values are shown.

We started by re-examining the profiles of the 200 and 220 reflections from a PMN–32%PT single crystal collected using a single avalanche photodiode point (Fig. 2 ▸). The electric field was applied using the system described previously by Wooldridge *et al.* (2012 ▸). The system allowed an alternating electric field of a variable frequency to be applied to the sample. The polarization–electric field (PE) hysteresis loops were acquired, and the diffraction pattern was collected at 40 points on this PE loop. This was a stroboscopic measurement since the time taken to collect a statistically meaningful diffraction pattern is 10–100 times longer than the time taken to measure the polarization. Fig. 2 ▸ shows the effect of the voltage changes on the structure; for simplicity only three of the 40 electric field values are shown for each reflection. These measurements were carried out on beamline 28 (XMaS) at the ESRF with the sample mounted on a six-circle diffractometer and the data being collected by a single avalanche photodiode (APD). The 200 and 220 reflections were modeled by fitting a single monoclinic phase with some preferred orientation induced by the electric field and by a two-phase model containing a monoclinic (*Pm*) and a tetragonal (*P*4*mm*) phase. It can be seen from an analysis of the fit quality demonstrated in Fig. 2 ▸ and the good figures of merit published by Wooldridge *et al.* (2012 ▸) that for each model there is an acceptable fit. Even with inclusion of the 222 peak together with 220 and 200 it was not possible to distinguish between the various models. As a consequence, we concluded that there were insufficient data to unambiguously solve this problem.

**Figure 3:**
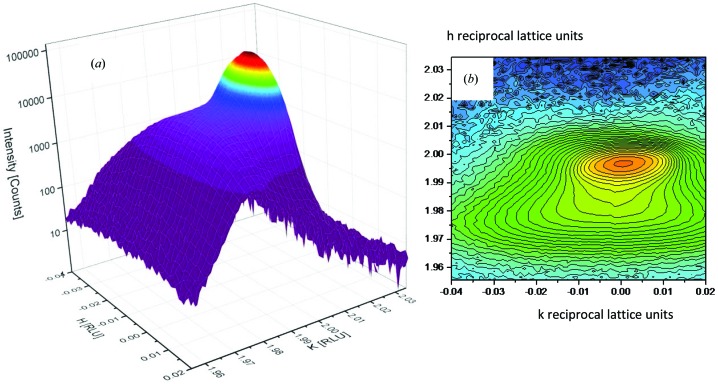
(*a*) The 200 reflection with the intensity on a log scale under an applied electric field of 1 kV mm^−1^. The peak shows a number of distortions including twinning (wide mosaic spread) and diffuse scattering streaks. The time taken for the scan was approximately 4 h. (*b*) The regions tending to twin below the main peak.

It was also clear from a complete three-dimensional reciprocal space map (Fig. 3 ▸) for the 200 reflection that diffuse scatter and a large mosaic spread/twinning were present (from observations of the ω scan profiles). Fig. 3 ▸ shows a surface contour map (*a*) and an area section plot (*b*) for the 200 reflection as mapped out by a single APD detector scanned over the pseudo-cubic reciprocal lattice node.

The diffuse streaks are particularly marked on the 200 reflection but were also seen to be present in other reflections. The mosaic spread was observed to be well over 5°, which was subsequently found to be mainly caused by twinning. Each three-dimensional reflection map took 3–4 h to collect using the single APD detector, making a full data acquisition involving more reflections impractical. The contour and area plots show signs of twinned regions, diffuse scatter and high backgrounds indicative of the need to scan a wider region of reciprocal space.

In order to resolve these measurement difficulties a Pilatus 2M area detector was used in conjunction with a system for the application of a static electric field in transmission geometry rather than reflection geometry. Transmission geometry required the use of smaller samples, which were produced as described in the previous section. The area detector allowed reciprocal space mapping of ∼40 reflections on a timescale of minutes compared with three reflections from the APD taking a whole night shift.

**Figure 4:**
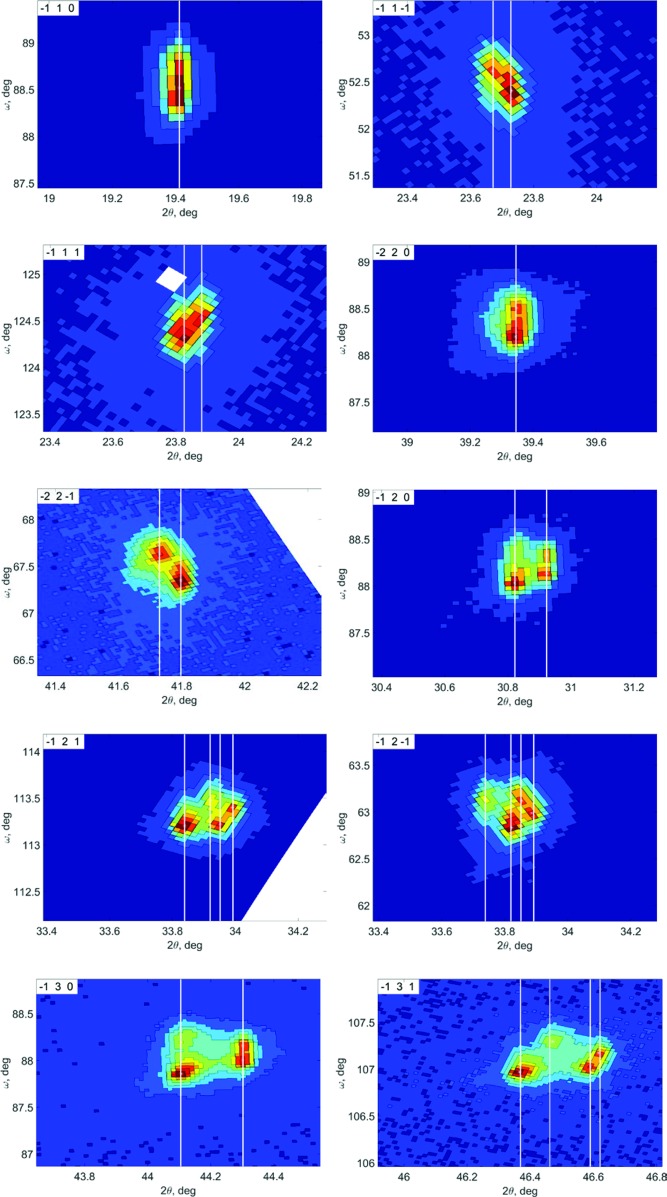
Ten reflections (*x* axis: 2θ; *y* axis: ω) measured from the PMN–33PT sample in transmission. The indices of reflections are marked in the left-top corner. The vertical white lines show the peak indexing.

The reciprocal space (ω *versus* 2θ) maps of ten different Bragg reflections under a statically applied 1300 V voltage (∼1850 V mm^−1^) are shown in Fig. 4 ▸. They exhibit clear splitting, corresponding to the formation of different pseudo-merohedral twin domains. This splitting was also observed in reflection mode, but it was difficult to make a robust interpretation with only two or three reflections. Although, some diffuse scattering can also be seen near the Bragg nodes, the detailed characterization of this diffuse background would require a separate experiment with longer exposure times.

We focused on the analysis of split Bragg reflections and the separation of the reflection composites along the 2θ (scattering angle) axis. This variable is directly connected to the length of the corresponding reciprocal lattice vector [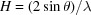, where λ is the X-ray wavelength]. We have adopted a strategy of data analysis that was previously introduced for the investigation of symmetry and lattice parameters in different perovskite-based twinned single crystals (Gorfman & Thomas, 2010 ▸; Datta *et al.*, 2009 ▸; Gorfman *et al.*, 2011 ▸). In the framework of this analysis, the number of peaks with different 2θ was used to assign the symmetry of the lattice (crystal system), while the separation along the 2θ axis was used to refine corresponding symmetry-allowed differences between the lattice parameters.

We assumed that the application of an external electric field along the pseudo-cubic *c* axis transforms the single-domain tetragonal *P*4*mm* to the multi-domain monoclinic *Pm* phase. Such symmetry lowering releases all the restrictions on the unit-cell lengths (*a*, *b* and *c*) and the unit-cell angle β. The shearing of the unit cell creates twin domains, which correspond to all the possible variants of the parent tetragonal axes *a* and *b*. Table 1 ▸ shows the number of different 2θ values in such twinned {*hkl*}_T_ reflections sets. The magnitude of peak separation is defined by the parameters |*b* − *a*| and β, which were refined against the observed data. The results are shown in Fig. 4 ▸, where the white vertical lines represent the simulated 2θ positions of the peaks.

**Figure 5:**
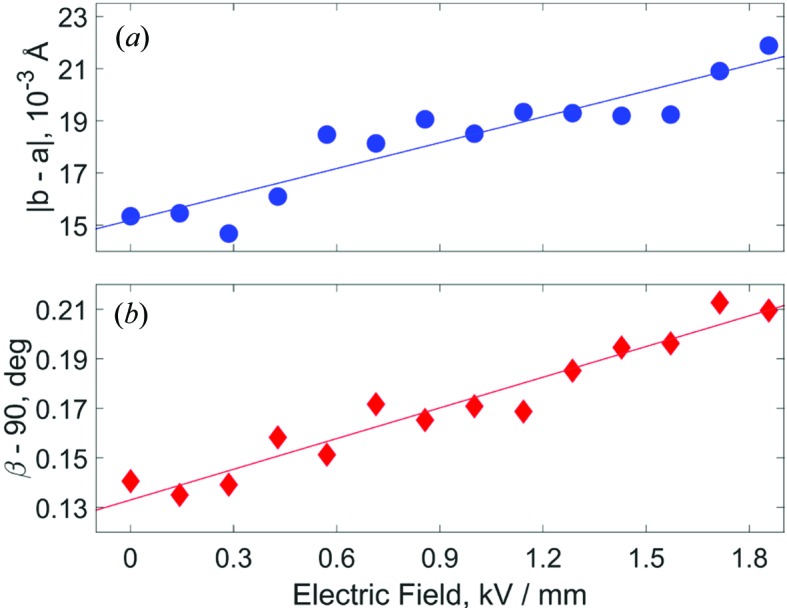
(*a*) The voltage dependence of lattice parameters 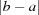 and (*b*) the refined monoclinic cell angle, β. Both parameters are displayed as a function of electric field.

Similar reciprocal space maps and splitting patterns were observed for the case of all the other voltages applied to the sample. These are deposited as supporting information. The evolution of the refined parameters 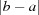 and β is shown in Fig. 5 ▸ as a function of applied electric field. We used the electric field dependences in Fig. 5 ▸ to extract information about the intrinsic piezoelectric coefficients. These are defined by the third-rank piezoelectric tensor (Nye, 1957 ▸) 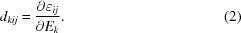
Here 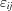 are the elements of the tensor of strain (related to the change of the lattice parameter) and 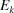 are the elements of the electric field vector. All the vector and tensor components are defined relative to the crystal physical Cartesian coordinate system {**e**_1_, **e**_2_, **e**_3_}, for which **e**_3_ || **c** and **e**_2_ || **b**. Because the electric field was parallel to the **c**/**e**_3_ axis, the data provide information about the 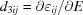 piezoelectric coefficients only. For the case of monoclinic distortion the strain tensor is given by 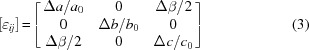
where 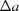, 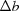 and 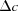 are the field-induced changes of the unit-cell lengths; 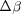 is the field-induced change of the monoclinic angle (expressed in radians); and 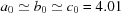
 Å are the undistorted lattice parameters. Using equations (2) and (3) and the linear regression coefficients in Fig. 5 ▸, we calculated that 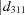 − 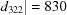
 pC N^−1^ and 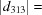 375 pC N^−1^. The previously re­ported macroscopically measured piezoelectric coefficient was of the order of 2500 pC N^−1^ (Luo *et al.*, 2000 ▸). Since our data are based on 2θ splitting rather than absolute 2θ values it is not possible to directly measure the absolute values for *d*_311_, *d*_322_ or *d*_333._ However, the value of |*d*_311_ − *d*_322_| which we can access is likely to have the same order of magnitude as the absolute values of *d*_311_ and *d*_322_. Therefore, we can conclude that the intrinsic piezoelectricy must make a significant contribution to the overall electromechanical response. The second contribution comes from a domain wall motion, which changes the volume ratio between differently strained ferroelastic domains and can be observed as an intensity redistribution between parts of the peak. Decoupling the intrinsic and extrinsic contributions is beyond the scope of this paper.

## 4. Discussion   

The use of the area detector demonstrably enables correct space-group determination, observation of microstructure evolution and measurement of diffuse scattering from complete reciprocal space maps. The combination of the system for sample mounting and detection of diffraction data described here with the setup for *in situ* dielectric spectroscopy (Wooldridge *et al.*, 2012 ▸) will be a useful way to determine complex ferroelectric structures as a function of applied electric field (voltage and frequency). If the experiment requires time correlation between field variation and detection of diffraction patterns, then the limiting factor will be the speed of the detector. Thus, for the Pilatus 2M detector used in the present experiment the maximum frequency is 20 Hz, the modern release of the same detector offers 300 Hz, and the next generation of area pixel detectors, for example, the Eiger X, offer 3000 Hz (http://www.dectris.com). Higher frequencies would still require a point detector.

The area detector system has been shown to deliver useful information by efficiently measuring diffuse scattering from noncrystalline or nonperiodic crystal structures. It is well known that diffuse scattering contains information about atomic and molecular correlated motion (Faxen, 1923 ▸; Welberry & Goossens, 2014 ▸) as well as static short-range order. The modeling of diffuse scattering is not easy because the short-range order is not governed by the crystal symmetry. The crystal properties that cause diffuse scatter can be manifold but include the following: phonon activity; thermal diffuse scattering; distortions of an otherwise ordered lattice due to defects (Huang scattering); static (or very slow) displacements of the ions from average positions; ionic substitution; stacking faults; domains; domain walls; atomic diffusion. In each case these can be static or dynamic. Two exposures of shorter and longer time can be employed to record both Bragg and diffuse components, respectively, if the dynamic range of the detector is not sufficient to see them both in one data collection. Although we have studied a crystal of the piezoelectric PMN–PT series, 67% Pb(Mg_1/3_Nb_2/3_)O_3_–33% PbTiO_3_ (PMN–33%PT), and have shown it to be of single phase with a complex twinned domain microstructure, the proposed approach is of general use when studying electrically active materials.

The use of an area detector implies a volume of raw data encoding intensity distribution in the reciprocal space as a function of electric field. Faster and smarter algorithms for handling large (ferroelectric specific) data sets will be required, including deconvolution of domain patterns and analysis of domains’ and domain walls’ contributions. These elements are still to be developed. As a first step, we propose to use two-dimensional ω–2θ mapping; the maps can be easily calculated from the detector images and serve as a tool to see the symmetry and lattice distortions in twinned crystals (Gorfman & Thomas, 2010 ▸; Datta *et al.*, 2009 ▸; Gorfman *et al.*, 2011 ▸). Here we show how this approach combined with electric field measurements quantifies intrinsic lattice contributions in the piezoelectric coefficients. A further development of the method could be three-dimensional mapping with recovery of the orientation and the volume of domains as a function of electric field. In combination with *in situ* dielectric spectroscopy full three-dimensional mapping of reciprocal space could become a very effective *in situ* synchrotron tool to study ferroelectric and piezoelectric materials.

## Supplementary Material

Movie showing how the structure changes when the field is increased for the -1 1 -1 reflection. DOI: 10.1107/S1600576716011341/kc5040sup1.wmv

Movie showing how the structure changes when the field is increased for the -1 1 1 reflection. DOI: 10.1107/S1600576716011341/kc5040sup2.wmv

Movie showing how the structure changes when the field is increased for the -2 2 0 reflection. DOI: 10.1107/S1600576716011341/kc5040sup3.wmv

Movie showing how the structure changes when the field is increased for the -2 2 -1 reflection. DOI: 10.1107/S1600576716011341/kc5040sup4.wmv

Movie showing how the structure changes when the field is increased for the -1 2 0 reflection. DOI: 10.1107/S1600576716011341/kc5040sup5.wmv

Movie showing how the structure changes when the field is increased for the -1 2 -1 reflection. DOI: 10.1107/S1600576716011341/kc5040sup6.wmv

Movie showing how the structure changes when the field is increased for the -1 2 1 reflection. DOI: 10.1107/S1600576716011341/kc5040sup7.wmv

Movie showing how the structure changes when the field is increased for the -1 3 0 reflection. DOI: 10.1107/S1600576716011341/kc5040sup8.wmv

Movie showing how the structure changes when the field is increased for the -1 3 -1 reflection. DOI: 10.1107/S1600576716011341/kc5040sup9.wmv

Supplementary figures. Reciprocal space maps as a function of applied field and temperature.. DOI: 10.1107/S1600576716011341/kc5040sup10.pdf

## Figures and Tables

**Table 1:** Reflection sets and corresponding reciprocal lattice vectors

Reflection set	Number of peaks with different 2θ	Reciprocal lattice vectors, with different length
{*hh*0}_T_	1	[*hh*0] 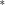
{*hhh*}_T_	2	[*hhh*] 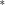 , [ 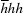 ] 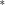
{*hk*0}_T_	2	[*hk*0] 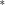 , [ 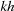 0] 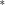
{*hkl*}_T_	4	[*hkl*] 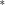 , [*khl*] 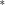 , [ 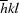 ] 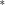 , [ 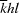 ] 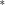
