# Supplementary material for: A rapid two-dimensional data collection system for the study of ferroelectric materials under external applied electric fields
Source: J Appl Crystallogr. 2016 Aug 16;49(Pt 5):1501–7. doi: 10.1107/S1600576716011341 (PMC5045728; doi:10.1107/S1600576716011341)
Supplement: Supplementary file 10 [file j-49-01501-sup10.pdf]

Supplementary Figure. Reciprocal space maps as a function of applied field and temperature. (*a*) 273 K and (*b*) 773 K.

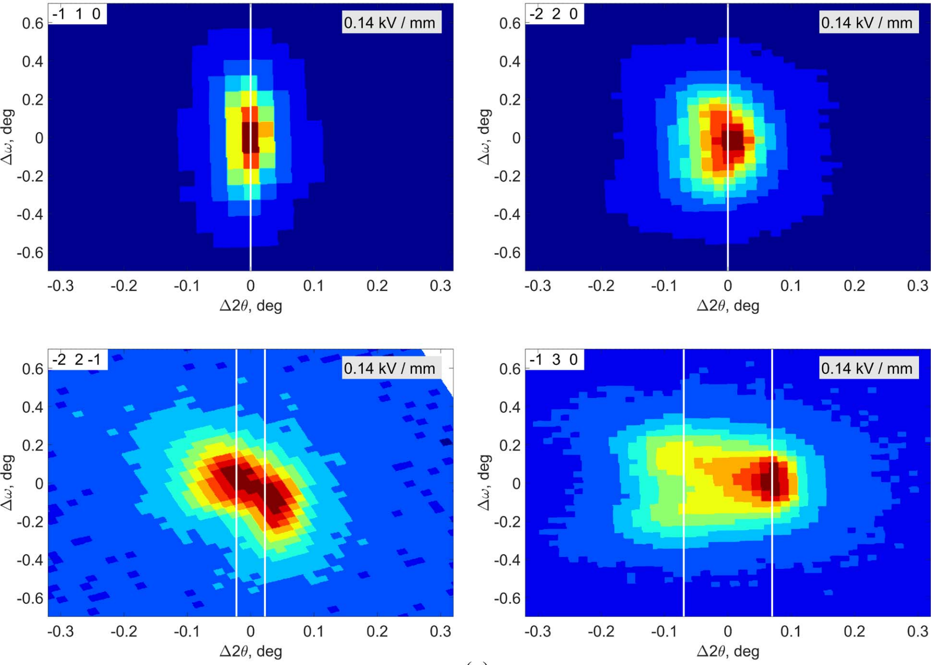

(*a*)

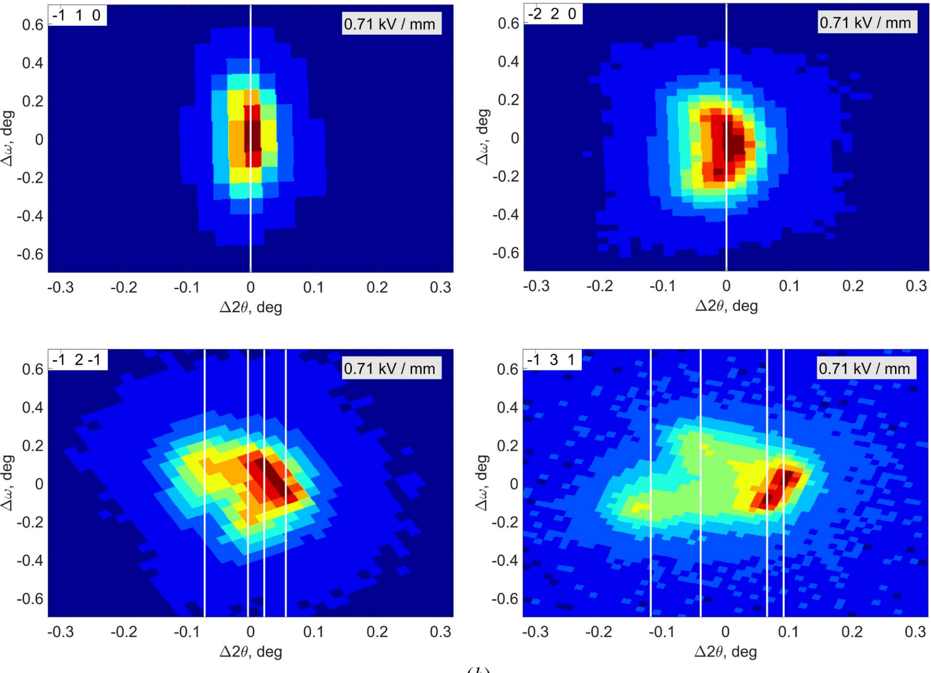

(*b*)
